# Giotto di Bondone (c. 1267-1337). St. Francis of Assisi Receiving the Stigmata (c. 1290)

**DOI:** 10.3201/eid0812.021200

**Published:** 2002-12

**Authors:** Polyxeni Potter

**Affiliations:** * Centers for Disease Control and Prevention, Atlanta, Georgia, USA

**Figure:**
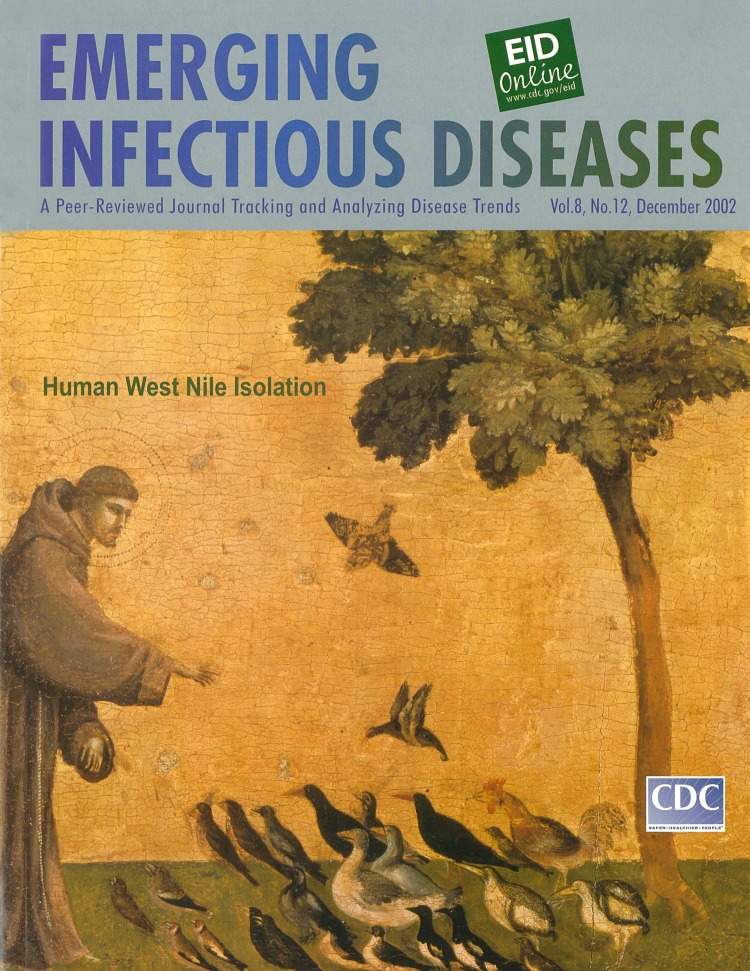
Giotto di Bondone (c. 1267-1337). St. Francis of Assisi Receiving the Stigmata (c. 1290). Tempera on wood, 313 cm x 163 cm. Musée du Louvre, Paris, France

Giotto di Bondone, founder of the Italian school of painting, was born in a village near Florence. Legend has it that Cimabue, the great master of the late 13th century, found Giotto herding sheep in the Italian countryside, noticed that the youth was drawing one of the sheep on a rock, and took him under his tutelage. Even though not much is known about Giotto’s life, his era of the great cathedrals was a time of artistic renaissance in Italy. While some argue that Giotto did not single-handedly create the remarkable artistic developments of his time, few dispute that these developments reached their peak in his work (1).

Giotto is considered the first painter in the history of Western art to place human figures within realistic surroundings. Moving away from Byzantine tradition, he introduced pictorial space, which strengthened figures, giving them three-dimensional force and structural importance. With a keen eye for detail, he painted characters in all walks of life, from peasants and townspeople, to mystics and popes. Giotto was recognized as an artist in his lifetime and was said to have “translated the art of painting from Greek to Latin” (2).

In 1296, Giotto was invited to paint the story of St. Francis of Assisi in 28 scenes for the famed San Francesco basilica in Pisa. This work, Giotto’s best, introduced the principles of actuality and reality in painting and was the earliest example of the Italian school. The painter drew from historical accounts of the life of St. Francis to create a likeness of the saint that was intense, realistic, and reminiscent of every day life—a complete departure from the stylistic and symbolic. The image of St. Francis was set in a topographic context highlighting the architectural and physical surroundings (1).

The rural scene featured on this cover of Emerging Infectious Diseases depicts St. Francis’ sermon to the birds. The scene lovingly merges the surroundings (the trees and the birds) in a harmonious composition of human activity (and divine presence) in perfect tune with nature—nature elevated in importance and set in the forefront. The monk, wearing a luminous halo, is engaged in a commonplace interaction with an odd collection of birds, humble creatures that nonetheless seem to appreciate the mysterious circumstance: the miracle of the stigmata (the marks, according to religious tradition, of Christ’s suffering).

In this depiction of St. Francis, Giotto’s genius lies not only in the skillful juxtaposition of the realism in the scene (contemporary attire, animals, trees, the monk) with notions of the miraculous, but also in the enlightened awareness of the world as a structured composite in which all the pieces, human or not, contribute to the integrity of the whole. Whether (as in the 13th century) they observe with rapt attention the stigmata of suffering or (as in our times) themselves carry the stigmata in the form of disease, the birds are clearly an integral part of the scene.
